# Mesenchymal stromal cell-derived secretome-based therapy for neurodegenerative diseases: overview of clinical trials

**DOI:** 10.1186/s13287-023-03264-0

**Published:** 2023-05-04

**Authors:** Maryam Ghasemi, Elham Roshandel, Mozhdeh Mohammadian, Behrouz Farhadihosseinabadi, Parvin Akbarzadehlaleh, Karim Shamsasenjan

**Affiliations:** 1 Student Research Committee, Tabriz University of Medical Sciences, Tabriz, Iran; 2 Stem Cell Research Center, Tabriz University of Medical Sciences, Tabriz, Iran; 3 Hematopoietic Stem Cell Research Center, Shahid Beheshti University of Medical Sciences, Tehran, Iran; 4 Department of Hematology, School of Medicine, Tarbiat Modares University (TMU), Tehran, Iran; 5 Breast Cancer Research Center, Motamed Cancer Institute, ACECR, Tehran, Iran; 6 Pharmaceutical Biotechnology Department, Pharmacy Faculty, Tabriz University of Medical Science, Tabriz, Iran

**Keywords:** Mesenchymal stem cell, Secretome, Culture media, Exosomes, Neurodegenerative disorders, Cell-free therapy

## Abstract

**Background:**

Over the past few years, mesenchymal stromal cells (MSCs) have attracted a great deal of scientific attention owing to their promising results in the treatment of incurable diseases. However, there are several concerns about their possible side effects after direct cell transplantation, including host immune response, time-consuming cell culture procedures, and the dependence of cell quality on the donor, which limit the application of MSCs in clinical trials. On the other hand, it is well accepted that the beneficial effects of MSCs are mediated by secretome rather than cell replacement. MSC secretome refers to a variety of bioactive molecules involved in different biological processes, specifically neuro-regeneration.

**Main body:**

Due to the limited ability of the central nervous system to compensate for neuronal loss and relieve disease progress, mesenchymal stem cell products may be used as a potential cure for central nervous system disorders. In the present study, the therapeutic effects of MSC secretome were reviewed and discussed the possible mechanisms in the three most prevalent central nervous system disorders, namely Alzheimer's disease, multiple sclerosis, and Parkinson's disease. The current work aimed to help discover new medicine for the mentioned complications.

**Conclusion:**

The use of MSC-derived secretomes in the treatment of the mentioned diseases has encouraging results, so it can be considered as a treatment option for which no treatment has been introduced so far.

## Background

Neurodegenerative disorders can be the result of an injury to the brain and spinal cord neurons. When the body is unable to replace damaged neurons, structural damage and function failure lead to neuronal death. Consequently, dementia, mental dysfunction, and movement problems occur in neurodegenerative diseases, such as Parkinson’s disease (PD) [1], Alzheimer’s disease (AD) [2], and multiple sclerosis (MS) [3]. Although the exact pathophysiology of neurodegenerative diseases is unclear, several studies have suggested that oxidative stress [4], environmental pollution [5], aging [6], infection [7], chemical exposure [8], and immune dysregulation [9] play a role in the accumulation of misfolded proteins (such as tau, amyloid-β, α-syncline) [10] over time. Different types of pharmacotherapies were developed to treat more common neurodegenerative disorders, like AD [11] and PD [12]; among them, acetylcholinesterase inhibitors (donepezil and rivastigmine) and N-methyl-D-aspartate (NMDA) receptor agonists were found to be more effective, especially for AD [13]. Regarding PD, only one drug, Xadago safinamide, has received FDA approval [14]. Despite their ability to alleviate symptoms, these drugs cannot stop the disease progression [15]. The available drugs for MS (glatiramer acetate, cladribine, natalizumab, mitoxantrone, and ocrelizumab) are ineffective in accelerating tissue repair [16]; therefore, attempts have been made to find new effective therapeutic strategies, such as stem cell therapy, to treat neurodegenerative diseases [17, 18].

Mesenchymal stromal cells (MSCs) are cells with the capacity for self-renewal and differentiation into various cell lineages [19]. The International Society of Cell Therapy (ISCT) states that MSCs must satisfy three criteria to be labeled as MSCs; these are adipogenesis, chondrogenesis, and osteogenesis; plastic adhesion property; and positivity of CD90, CD105, and CD73 surface markers along with negativity for CD45, CD19, CD79a, CD34, and human leukocyte antigen (HLA)-DR surface markers. In contrast to other stem cell groups, the expression of CD34 is believed to be challenging [20], and no specific marker has been developed to identify MSCs [21]. Mesenchymal stromal cells can be derived from several sources, including bone marrow, adipose tissue, dental tissues, placenta, umbilical cord blood, Wharton's jelly, and the brain [22]. Despite meeting the abovementioned criteria, these mesenchymal stromal cells from different sources differ in several characteristics, such as their ability to differentiate into specific cell lineages, their cytokine secretion profiles, and surface markers [23, 24].

More than 2000 patients suffering from different stages of neurodegenerative diseases received MSCs, and most of them achieved promising results [25]. Nonetheless, there are several concerns about MSC-based cell therapy, including heterogeneity, potential side effects after allogeneic MSC transplantation, and the difficulties in choosing proper donors. Heterogeneity could be due to differences in MSC sources and cell culture methods [26, 27]. This fact makes it difficult to compare results from clinical trials that have chosen different sources and methods. From another point of view, it can be considered a favorable option in applying MSC to treat different conditions by manipulating the culture medium and choosing the appropriate source to achieve the optimum results.

Another concern is the host immune system's reaction after transplanting MSCs. The use of autogenic MSCs is considered an option to avoid this complication; however, it has certain limitations since the potential therapeutic effects of MSCs depend on the donor’s age and health [28]. Previous studies have revealed that MSCs obtained from patients with obesity [29] and inflammatory diseases did not have normal differentiation and proliferation capacity; therefore, they could not produce the expected therapeutic effects [30]. The last concern is about the growing data suggesting that MSC therapy may be ineffective, in particular for neural disorders. One reason may be the weak and transient benefit of MSC therapy in studies of central nerves system (CNS) disorders, such as ALS [31], MS [32], and stroke [33]. It could be the result of an ineffective transplantation method. After intravenous (IV) transfusion, MSCs are trapped in some organs, mostly in the lungs [34]. In most cases, they cannot cross the blood–brain barrier successfully. Applying a novel method of MSCs transplantation, such as intralesional, intranasal, intra-arterial, or the use of MSC’s secretome, solves this issue [35].

The proteins released by cells are a straightforward definition of the secretome. Secretomes derived from MSCs include soluble (cytokines and chemokine) and insoluble (extra vesicles) factors [36]. MSC-derived secretomes are composed of a protein-soluble fraction, like growth factors and cytokines, and a vesicular fraction, composed of microvesicles and exosomes. Insoluble components are released into the extracellular environment and participate in trafficking, adhesion, and endocrine signaling [37]. Exosomes' cargo consists of three categories: I. proteins, which include signaling peptides, heat-shock proteins, vesicular transport proteins, signal transduction proteins, cytoskeletal proteins, and cell metabolism enzymes [38]; II. lipids, which include ceramides, cholesterols, phosphatidylserines, and sphingomyelins; III. nucleic acids which include genomic DNA, mitochondrial DNA (mtDNA), transfer RNAs (tRNAs) [39], messenger RNAs (mRNAs), ribosomal RNAs (rRNAs), complementary DNA (cDNAs), and small and long non-coding RNA [40]. Numerous recent studies have highlighted the beneficial therapeutic effects associated with MSC transplantation to the CNS due to its higher paracrine activity than its cell differentiation capacity [41]. The presence of immune regulatory and neurotrophic factors in MSCs’ secretome plays a crucial role in their desirable effects on CNS disorders. Some of these neurotrophic factors can increase neuronal proliferation and survival, such as nerve growth factor (NGF), glial cell-derived neurotrophic factor (GDNF), brain-derived neurotrophic factor (BDNF), neurotrophin-3 (NT-3), NT-4, vascular endothelial growth factor (VEGF), hepatocyte growth factor (HGF), fibroblast growth factor (FGF), pigment epithelium-derived factor (PEDF), insulin-like growth factor (IGF)-1, IGF2, transforming growth factor-beta 1 (TGF-β1), interleukin (IL)-6, pigment epithelium-derived factor (PEDF), DJ-1, and cystatin-C (Cys-C) [42].

To obtain MSC-derived exosomes, first, MSCs cultivated in an exosome production medium, and then the supernatant undergoes the isolation methods. The lack of standard isolation and purification methods is the main obstacle in routing exosomes into the translational clinical application. Although exosome isolation is conventionally performed using ultracentrifugation (differential ultracentrifugation, density gradient centrifugation), the gold-standard method, other techniques have been developed to overcome the ultracentrifugation limitations. The alternative isolation techniques are based on isolation by size (ultrafiltration, sequential filtration, exosome isolation kits, size exclusion chromatography, flow field-flow fractionation, hydrostatic filtration dialysis), immunoaffinity capture (enzyme-linked immunosorbent assay, magneto-immunoprecipitation), exosome precipitation (polyethylene glycol precipitation, lectin induced agglutination), and microfluidic techniques (acoustic nano-filter, immuno-based microfluidic isolation). Each isolation method has some advantages and disadvantages. For instance, immunoprecipitation yields the most efficient recovery rate, while the acoustic nano-filter method gives the highest purity and requires the least sample volume and time. The exosome analysis is performed via different methods based on physical characteristics (electron microscopy, dynamic light scattering, nanoparticle tracking analysis, tunable resistive pulse sensing), chemical, biochemical, and compositional characteristics (immunodetection methods such as flow cytometry and western blotting, thermophoretic profiling, and mass spectrometry-based proteomic analysis) [43].

In this study, the effects of MSCs on cells on CNS injury were reviewed. The focus of the study was on secretome in three common diseases, namely Alzheimer’s disease, Parkinson’s disease, and MS. This was done to explore the therapeutic horizons concerning these conditions.

## Main text

### Mechanism of function

Different mechanisms are considered to explain the beneficial effects of MSC transplantation [44, 45]. In vivo studies have shown that autologous or exogenous MSCs could modify tissue structure and function by migrating to damaged areas, which involves several steps. Although the exact homing mechanism is not fully understood, there is a hypothesis based on its similarity to leukocyte migration via integrins, selectins, adhesion molecules (like VCAM-1), and G-protein signaling pathways [46]. Upon migration to the CNS, MSCs interact with surrounding tissues. This leads to neuronal differentiation and secretion of cytokines and growth factors involved in various mechanisms, such as neuroprotection, immunoregulation, angiogenesis, and inhibiting neuronal apoptosis [47]. Despite the beneficial features of MSCs, they show limited differentiation capacity compared to other types of stem cells, like embryonic pluripotent stem cells. It is not entirely accepted that MSCs can replace all lost neurons through neural differentiation. Therefore, improvement in neuronal survival following MSC administration is possibly due to the secretion of various neurotrophic factors by MSCs rather than differentiation. Secretion of neurotrophic factors can be increased by manipulating culture media [48]. Some priming protocols have been assessed in MSC culture media to induce particular alterations, leading to desirable changes [49]. For instance, Redondo-Castro et al. reported that preconditioning treatments of MSCs with inflammatory cytokines, like IL-1, prime them for a neurotrophic phenotype to release secretomes containing neurotrophic and anti-inflammatory cargos in the culture media supernatant []. Findings indicated that inducing MSCs to overexpress neurotrophic factors like GDNF and BDNF resulted in promising outcomes in PD and AD mouse models [50]. Moreover, using dynamic culturing conditions in computer-controlled bioreactors induces MSCs to produce a higher amount of neurotrophic factor, leading to a more effective cocktail for therapeutic applications in neurodegenerative disease [51, 52].

Immune dysfunctionality has been introduced as one of the main causes of neurodegenerative diseases such as AD, PD, and MS. In this light, glial cells’ hyperactivity and immune cells’ infiltration into the CNS lead to disease progression [3]. But the MSCs’ capacity to affect the proliferation and activation of all types of immune cells is another promising feature that assures their efficacy in neurodegenerative disorders [53]. MSCs are not immunosuppressive by nature, but they need a specific cytokine profile [54]. However, the exact mechanism is still under question. Some reports have indicated that it occurs directly through cell-cell contact and secreting soluble factors [55]. MSCs interrupt three pivotal phases of the immune response: antigen presentation, T cell activation and proliferation, and effectors’ responses [53], through modulating the behavior of macrophages, dendritic cells (DCs), natural killer cells (NK cells), B cells, and T cells. It happens mostly through secreting certain molecules, including indoleamine 2,3-dioxygenase (IDO), prostaglandin E2 (PGE2), TGF-β1, HLA-G5, IL-10, and IL-6 [56]. Besides, the mediators secreted by MSCs comprise various cytokines and growth factors that mostly play an immunomodulatory role in the cell microenvironment. They also actively produce neurotrophic factors like BDNF, GDNF, VEGF, NT-3, NGF, and IGF, which induce endogenous neurogenesis and raise neuronal survival. Numerous studies have confirmed the neuro-protective effects of these growth factors [57, 58]. GDNF, as a well-known trophic factor, was reported to exhibit remarkable neuroprotective properties [59]. BDNF is the main promoting agent to axonal outgrowth in the CNS, since with its removal from the secretome, the effect was not observed anymore [60]. Moreover, several papers have introduced VEGF as another critical factor, which induces explicitly axonal outgrowth [61, 62]. This result was confirmed by Zhou et al. as they promoted neurogenesis and functional recovery following VEGF and BDNF co-overexpressed MSC administration to the cerebral ischemia model [63].

MSCs can impair antigen presentation by DCs through reducing surface markers such as CD-11c, major histocompatibility complex (MHC)-class II, and CD83, indirectly leading to the inhibition of adaptive immune cell activation [64]. Cross-talk with macrophages, which play an imperative role in CNS inflammation under the high level of inflammatory cytokines (IFN-γ and TNF-α), polarizes them toward anti-inflammatory phenotype (M2), unlike pro-inflammatory (M1), which can contribute to tissue regeneration by increasing arginase-1 and IL-10 levels [65]. Microglial cells, which are resident macrophages in CNS tissue, can cause inflammation (by secreting IL-6, NO, TNF-α, and IL-1β) and are related to different pathogens of neurodegenerative disorders [66]. MSCs, through secretion of TNF-α, stimulated protein 6 (TSG-6), suppress microglia activation [67], and exert M1 to M2 switch by activating the CX3CL1/CX3CR1 signaling pathway in these cells [68].

MSCs suppress lymphocytes via three vital mechanisms: firstly, reducing the proliferation of both T [69] and B [70] lymphocytes by arresting the cell cycle in the G0/G1 phase even after exposure to allogenic cells; secondly, reducing IFN-γ and IL-17 production by polarizing the Th0 toward Th2 shift rather than that toward Th1 and Th17; and lastly, indirect induction of Treg generation [71]. It has been shown that MSCs exert further Treg immune suppression activity, probably via IL-10 secretion [72]. It could be a reassuring option for treating CNS disorders with autoimmune origins, such as MS.

Other secretory products, like HLA-G5, TGF-β1, IDO, and PGE2, suppress the proliferation, cytokine secretion, and cytotoxicity of NK cells. They also inhibit the activity of NK cells as a bridge between innate and adaptive immune responses [73].

To sum up, the beneficial effect of MSCs on neurodegenerative disorders is mostly attributed to the secretion of several cytokines and growth factors involved in immune regulation and neuroprotection.

### Secretome-based therapy in neurodegenerative diseases

Gnocchi first discovered the therapy based on MSC secretome in 2005 [44], which attracted scientific attention owing to addressing several concerns about the side effects following allogenic or autologous MSC transplantation. In addition to the risk of immune response and tumor genesis, culturing MSCs takes too much time for cell proliferation and may be associated with undesirable differentiation [45, 74]. On the other hand, MSC secretomes can be easily collected from commercial culture media without any invasive process [75]. Furthermore, in various types of diseases related to the damaged structure of different brain parts, administering culture media is effective. These reasons, along with the high prevalence of central nervous system disorders and their low regenerative potential, make these diseases one of the critical targets for MSC secretome-based therapy. Numerous studies have verified the neuroprotective and neurotrophic effects of MSC secretome based on this hypothesis [76]. Teixeira et al. reported that just a single administration of MSC secretome, even without cell transplantation, increased endogenous neuronal differentiation in the hippocampus region [77]. MSC-derived secretomes are flexible according to the state and site of the injury [78]. Therefore, they could be regulated by various pathological conditions similar to what may be observed in different CNS disorders.

In the following sections, the studies conducted on the effect of secretome-based therapies in the three main neurodegenerative diseases, including AD, PD, and MS, are reviewed (Table 1).

**Table 1:** Therapeutic effects of MSC in animal models of neurodegenerative diseases

Disease	Tissue source of MSC	Therapeutic effects	Refs.	Year
AD	Rat AD	Reduced oxidative stress; alleviated cognitive impairment; promoted neurogenesis; increased the neuroblasts numbers	[152]	2014
	Human UCB	Improved endogenous hippocampal neurogenesis and synaptic activity	[153]	2015
	Human WJ	Improved the spatial learning; mitigated memory decline; reduced Aβ soluble levels and its deposition	[154]	2016
	Human WJ and UCB	Induced neuronal development and neurite outgrowth	[155]	2016
	Human WJ	Reduces the accumulation of ubiquitin-conjugated proteins	[156	2017
	Human AM	Reduced amyloid-β peptide deposition; rescued spatial learning and memory	[157]	2017
	Mouse BM	Reduced Aβ plaque size	[158]	2017
	Human UCB	Mitigated Aβ-induced synaptic dysfunction	[159]	2018
	Human MenSCs	Improved spatial learning and memory; mitigated amyloid plaques; increased Aβ degrading enzymes; modulated panel of proinflammatory cytokines	[160]	2018
	Mouse BM	Improved cognitive impairment	[161]	2020
	Mouse BM	Reduction β-amyloid deposits	[162]	2020
	Human UCB	Improved the spatial learning; improved memory impairment	[163]	2020
	Rat BM	Improved cognitive impairment	[164 ]	2020
	Mouse BM	Memory recovery; reduced neuro-inflammation; decreased brain amyloidosis; increased neuronal density in cortex and hippocampus; diminished hippocampal shrinkage	[165]	2021
	Human WJ	Reduced cell death; reduced ubiquitin conjugate levels; reduced Aβ levels	[166]	2021
MS	Mouse BM	Improved clinical score	[167]	2015
	Mouse BM	Decreased T helper-17 activation and function	[168]	2015
	Mouse BM	Increased remyelination; decreased demyelination and apoptosis	[169]	2015
	Mouse AD	Reduced disease severity, inflammatory cell infiltration, and demyelination	[170]	2017
	Rat BM	Increased clinical score; declined inflammation	[171]	2017
	Mouse BM	Decreased vascular alteration of vessels, myelin, and neuronal damage	[172]	2017
	Human BM	Diminished demyelination in corpus callosum	[173]	2018
	Mouse BM	Improved therapeutic function	[174]	2018
	Rat BM	Differentiation of oligodendrocyte precursor cells	[175]	2019
	Mouse BM	Improved remyelination; declined microgliosis and astrocytosis	[177]	2019
	Mouse BM	Increased M2 phenotype macrophage; decreased M1 phenotype macrophage	[177]	2019
	Mouse BM	Reduced inflammatory infiltration and demyelination of spinal cord	[178]	2020
	Mouse BM	Increased neurobehavioral outcomes; reduced blood–brain barrier disruption, inflammatory infiltration, and demyelination in spinal cord	[179]	2020
	Human BM	Increased retinal ganglion cell function and motor sensory impairment	[180]	2020
PD	Rat BM	Neuro-protective effects on dopaminergic neurons	[181]	2008
	Rat BM	Partial rescue of dopaminergic pathway	[182]	2008
	Mouse BM	Neuro-protective effects on dopaminergic neurons; reduced blood–brain barrier damage; downregulation of neuro-inflammation	[183]	2009
	Rat BM	Improved viability of striatal/nigral dopaminergic terminals concomitant	[184]	2010
	Human endometrial	Improved dopamine production	[185]	2011
	Human BM	No effect on motor impairment	[186]	2015
	Human endometrial	Enhanced dopamine metabolite concentrations	[187]	2015
	Rat BM	Restored rotational behavior	[188]	2015
	Rat BM	Improved locomotor functions	[189]	2017
	Rat BM	Differentiation into nestin- and neuron-specific enolase-positive cells	[190]	2017
	Human BM	Reduced pro-inflammatory cytokines; restored behavioral function	[191]	2020
	Human BM	Down-regulated pro-inflammatory cytokines; stimulated antioxidant enzymes	[193]	2020
	Human AT	Induced alteration in dopamine transporter expression; promoted neurotrophic factors	[192]	2020
	Human WJ	Restored dopaminergic neurons; enhanced levels of neurotrophic factors	[194]	2020

*AD* Alzheimer’s disease, *MS* Multiple sclerosis, *PD* Parkinson’s disease, *BM-MSCs* Bone marrow-derived mesenchymal stem cells, *WJ-MSCs* Wharton’s Jelly mesenchymal stem cells, *UCB-MSCs* Umbilical cord blood-derived mesenchymal stem cells, *MenSCs* Menstrual blood-derived mesenchymal stem cells, *AD-MSCs* Adipose tissue-derived mesenchymal stem cells, *AM-MSCs* Amniotic mesenchymal stem cells

### Alzheimer's disease (AD)

AD is a prevalent chronic disease of the central nervous system with multifactorial and relatively complex pathology. AD patients suffer from the gradual loss of brain abilities such as memory and cognitive function. It has been estimated that approximately 131 million people, even those under the age of 65, will be affected until 2050 worldwide.

AD was primarily discovered in 1907 by Alzheimer, a neuropathologist specialist [79], and has since been divided into sporadic (sAD) and familial (fAD) groups. In the fAD group, three common gene mutations were reported, namely presenilin 1 (PSEN1), presenilin 2 (PSEN2) β, and precursor protein (APP) [80]. In sAD, apolipoprotein E (APOE), nutrition, lifestyle, and aging are known as major risk factors. However, the exact underlying mechanism remains ambiguous. Any efficient drug or curative method has been investigated. Although some current treatment methods, including cholinesterase blockers and NMDA receptor antagonists, are effective in the early stages of the disease, they are not highly efficient after a while due to a high number of neuronal losses in the hippocampus, the inability of drugs to cross the blood–brain barrier (BBB), and the increasing side effects following long-term use [81].

The main pathological features in both fAD and sAD are chronic accumulation of amyloid-beta (Aβ) plaque and neurofibrillary tangles (NFT) enriched by hyperphosphorylated protein tau [74]. This accumulation leads to the hyperactivation of microglia (MG) and astrocytes (AC) as the most involved immune cells in AD pathology [82]. Brain neurons of AD patients are permanently susceptible to inflammatory conditions induced by these immune cells. Although opinions about the role of MG and AC are controversial, most researchers consider them immunostimulators after exposure to Aβ plaques by their toll-like surface receptors (TLRs) (TLR 2, TLR4, TLR6, and TLR9) and CD markers (CD36, CD14, and CD47) [83, 84]. Expression of these TLRs in different cells has been suggested as the initiation of the inflammatory response. Thus, increased TLR2 levels are considered a diagnostic marker for activated MGs [85], and TLR4 overexpressing neurons are more prone to degradation after releasing IL-1B, TNFα, and IL-17 [86]. Modulating the TLRs' stimulation would be a critical step to control MG activation and AC status. In this context, one paper showed that hypoxia-preconditioned adipocyte-derived MSC secretome injection to AD mouse models decreased TLR2 and TLR4 expression, similar to astrocyte inflammatory cytokines, such as IL-1 and TNFα, which increase hippocampus neuronal survival [87].

Secreting inflammatory cytokines by activated MGs and ACs plays an important role in disease progression, neurotoxicity, and cognitive dysfunction [88]. Using IL-17 neutralizing antibodies improved memory function in an animal model of AD by preventing pro-inflammatory mediators [89]. On the other hand, some types of secretory products are crucial factors that cause resistance to the accumulation of AB plaque in the brain. An interesting study examined the expression of inflammatory and anti-inflammatory cytokines in the brains of the resistance group. Comparing the control and AD groups showed that they expressed different cytokines, which prevented neurodegeneration and dementia despite the presence of AB plaque [90]. The resistance groups differed in the number of Aβ plaques and NTFs. Higher levels of IL-6, IL-1ra, IL-13, and IL-4 belonged to the highest rate group (HP), whereas the dominant cytokines in the lowest rate group (LP) were IL-10, IL-6, and IP10. Among them, IL-4, IL-13, and IL-10 shifted the activated MG to M2 form to modulate the inflammatory response. The M1 phenotype massively releases inflammatory cytokines, which deteriorate CNS damage [91]. Conversely, the phenotypical exchange of glial cells in AD has been reported in favor of the inflammatory process. This has been reported during aging, diabetes, obesity, and some other mentioned risk factors associated with immune disturbances [92].

**Fig. 1:**
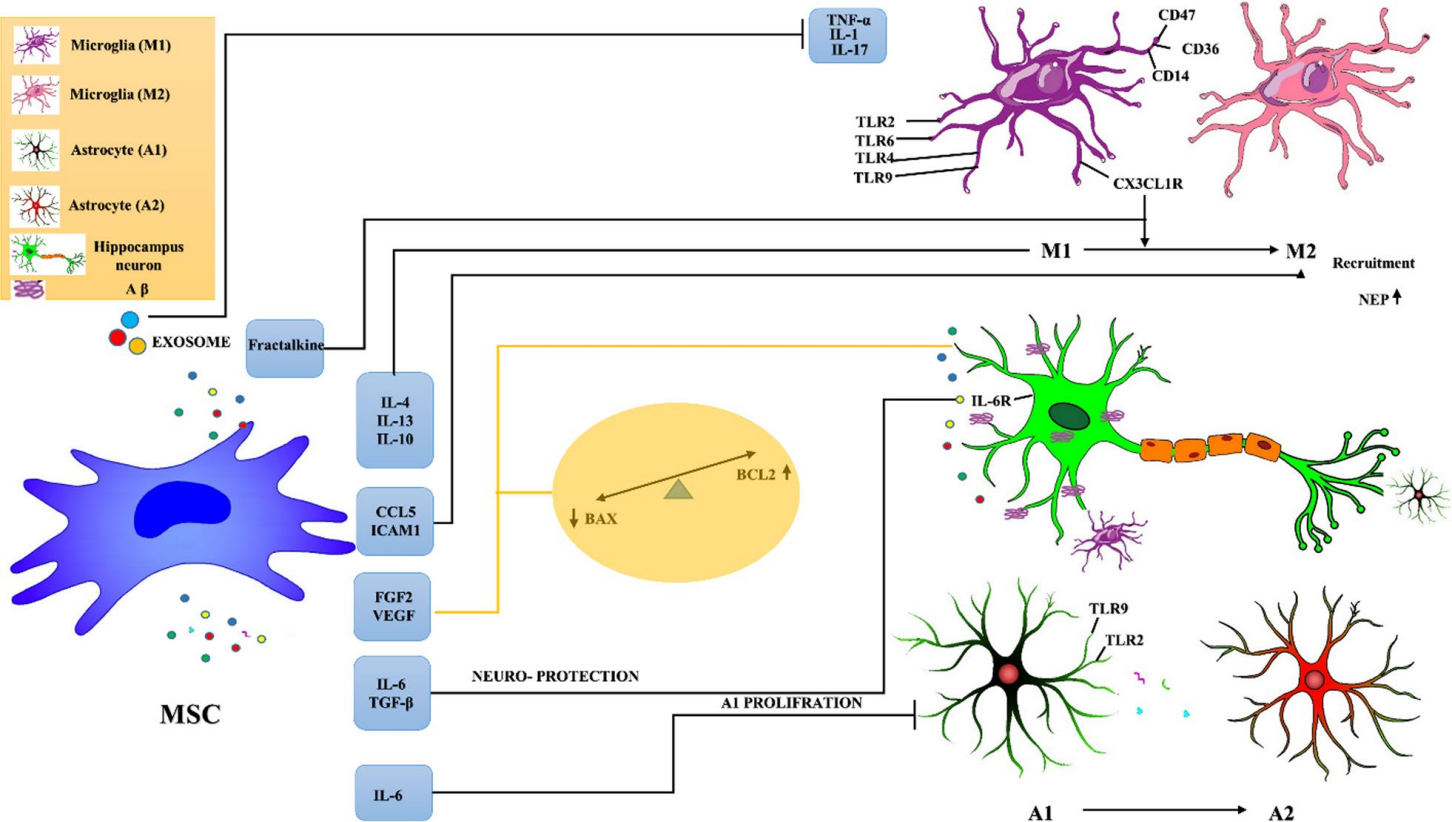
Graphical summary of different mechanisms conducted by MSCs secretome to hippocampal neuron protection. MSC products ameliorate neurodegeneration related to (A1) astrocyte and (M1) microglia, two major immune cells involved in the pathogenesis of Alzheimer’s disease. CX3CL1, IL- 4, IL-13, and IL-10 induced MGs switching from harmful phenotype M1 to protective form M2. Exosomes decrease the expression levels of IL-1a, IL-1β, and TNF-α; thus, MSCs can modulate the excessive inflammatory responses. The elevated secretion of CCL5 and ICAM-1 induces Aβ plaque degradation by increasing the level of protease enzyme (NEP). VEGF and FGF-2 can decrease neuronal apoptosis by changing the BAX/BCL-2 balance in favor of cell survival. IL: interleukin, TGF-β: transforming growth factor-beta, Fractalkine R: fractalkine receptor, TLR: Toll-like receptor, NEP: neprilysin, Aβ: amyloid beta, BAX: BCL-2-associated X, apoptosis regulator, BCL-2: B cell CLL/lymphoma 2. The images depicted in the figure are designed by authors

The MSC secretome comprises an array of bioactive molecules that ameliorate AD-related symptoms through different mechanisms (Fig. 1).

Transplantation of hypoxia-preconditioned MSC-exosomes to app/PS1 mouse models induced phenotypic exchanges in the lesion area by decreasing the expression levels of IL-1a, IL-1β, and TNF-α and increasing IL-4 and IL-10; thus, MSCs can modulate the excessive inflammatory responses caused by M1 MGs. Additionally, they suppressed the activation of astrocytes and decreased AB plaque deposition, leading to improved memory deficits and cognitive AD-related disorders [88]. In line with previous studies, MSC-derived CX3CL1 induced MGs switch from harmful phenotype M1 to protective form M2 through the fractalkine receptor, thereby protecting neurons against destructive effects following inflammatory cytokines release [93].

A high concentration of inflammatory products is commonly believed a sign of MGs and ACs shifting to a harmful form that compromises Aβ clearance. However, it seems that it could not be applied to all inflammatory cytokines [94]. Multiple studies have found TGF-β, IL-6, and other inflammatory cytokines accumulating around Aβ plaques in the brains of AD patients [2]. According to researchers, these two cytokines protect neurons by increasing plaque clearance, which results in improved cognitive and behavioral disorders [29]. IL-6, a pleiotropic cytokine expressed at a high rate in both resistance groups (HP and IP), was reported to be protective and increase neuronal survival. Contrary to normal conditions, IL-6 concentration was shown to be significantly increased during disease progression [95]. However, data in this context are controversial since IL-6, as a well-known inflammatory mediator, might either initiate a harmful cascade leading to cerebral damage or facilitate the healing process via raising angiogenesis and other protective mechanisms [96]. In this regard, Yang et al. indicated reduced autophagy in hippocampal neurons induced by endogenous MSC-derived IL-6 by inhibiting the AMPK/mTOR pathway via the gp130-IL-6R receptor complex. They revealed that MSC-derived IL-6 significantly decreases Beclin 1 and LC3 II, autophagy-associated proteins (Fig. 97) [98]. It was also reported that this cytokine could decrease the proliferation of activated astrocytes by affecting the AMPK/mTOR signaling pathway [99]. Further investigation is needed to clarify the reciprocal role of inflammatory cytokines in AD.

**Fig. 2:**
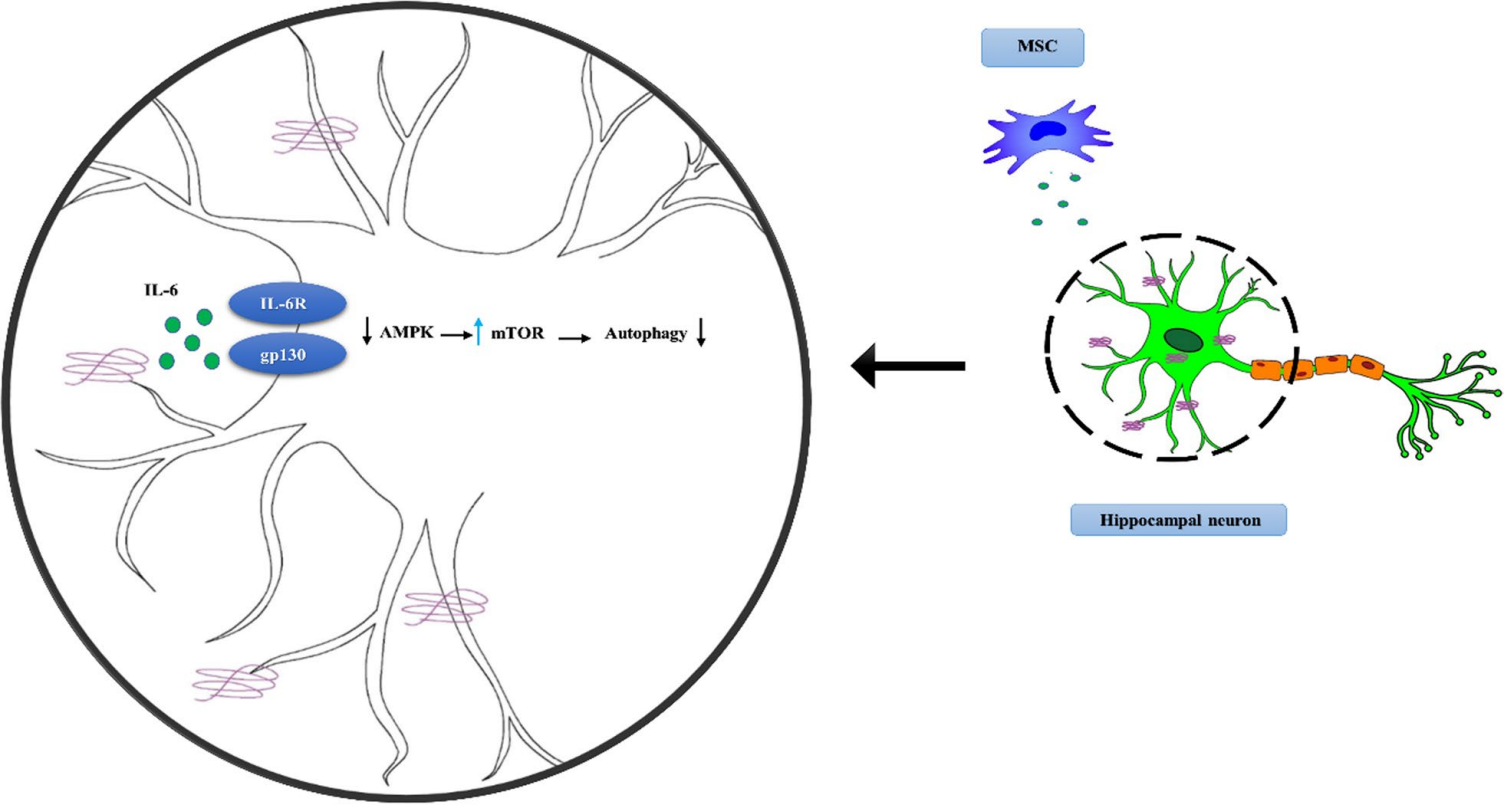
MSC-derived IL-6 suppresses the autophagy pathway in injured hippocampal neurons in Alzheimer’s disease through the downregulation of the AMPK (mitogen-activated protein kinase) signaling pathway. The AMPK signaling pathway is a downstream target of IL-6. IL-6 binds to the gp130-IL-6R receptor complex and induces a downregulation in the AMPK signaling pathway. IL-6 can induce mTOR, a pivotal factor in the autophagic signaling pathway, in an AMPK-dependent and STAT3-independent manner, leading to decreasing autophagy-associated proteins like Beclin 1 and LC3 II. The images depicted in the figure are designed by authors

Lee et al. injected bone marrow (BM)-derived MSCs into the hippocampal region of AD mice and realized that BM-derived MSCs could recruit activated MG cells to the inflamed area after exposure to Aβ deposition. The elevated secretion of CCL5, a chemoattractant factor, induced the recruitment of alternatively activated MG. Alternatively activated MGs attenuated memory impairment and decreased Aβ plaques through the secretion of IL-4 and neprilysin (NEP) [100]. Several beneficial effects of IL-4 were previously reported in AD, including improved spatial learning and increased Aβ degradation in phagocytic cells [101].

MSCs secretome can indirectly contribute to Aβ plaque degradation through the NEP enzyme. Neprilysin is a well-known protease enzyme that has an inverse relationship with the Aβ level. Recently, it has become a new target in the search for novel AD therapies. Cultured MSC-derived secretomes have the potential to regulate neprilysin’s activity. In vitro studies have revealed that MSC-derived soluble intracellular adhesion molecule-1 (ICAM-1) [99] and CCL5 [100] induce Aβ plaque degradation by increasing the level of NEP through alternative activation of MG cells. However, there is little knowledge about this enzyme and it needs further investigation.

MSC secretome not only contains NEP, but can also decrease the toxic effects and neuronal apoptosis caused by Aβ. MSC-derived secretome significantly reduced neuronal apoptosis by changing the BAX/BCL-2 balance in favor of cell survival. Aβ plaques induce neuronal apoptosis either directly by upregulating the Bax protein [102] or indirectly through increasing ROS-induced oxidative stress [103]. Dental pulp stem cell (DPSC)-derived secretomes can counteract Bax expression in neuroblastoma cell lines through upregulation of the anti-apoptotic protein Bcl-2. Scientists believed that it could be related to the expression of RANTES, Fractalkine, VEGF, and fibroblast growth factor-2 (FGF-2), which can promote Bcl-2 expression. Interestingly, it has also been proven that DPSCs secrete these cytokines and growth factors at higher concentrations than other common sources of MSC, like bone marrow and adipocytes [76]. Based on the aforementioned data, manipulating some factors aiming to upregulate Bcl-2 expression would be a promising strategy to develop more efficient therapies.

In summary, AD neuropathology is a relatively complex disease and requires multi-target therapy approaches. The use of MSCs with neuroprotection potential, potent immune modulation, and anti-amyloidogenic activities could be considered a multi-approach alternative for AD model therapy. MSC secretome immunomodulatory properties are partially regulated by MG and AC activity, either by regulating their proliferation potential and phenotypic changes from neurodegenerative to neuroprotective states, or by regulating TLR expression. However, further clinical trials are required to explain which one is precisely responsible for the observed therapeutic outcomes and optimize this option.

### Multiple sclerosis (MS)

MS is one of the prevalent neurodegenerative disorders affecting approximately 2.5 million people aged 20–40 years [103]. It is caused by the immune system attack through T helper reactive cells (TCD4 +) and antibodies against the lipoprotein of nerve fibers, due to genetic and environmental backgrounds. Chronic degeneration of myelin sheath leads to a progressive disability in patients. MS is divided into three stages according to clinical symptoms [104]. The most common type is relapse-remitting (RR) MS which occurs at an approximate rate of 85%-90% and is characterized by exacerbation and relative remission cycles [105]. The second phase is defined as "secondary progressive" (SP) and characterized by aggravated symptoms without remission periods, affecting 50–60% of MS patients. The third phase is defined as "primary progressive" (PP) and presented in about 15% of MS patients and is characterized by more severe clinical symptoms and chronic progressive disability with or without exacerbation episodes. Both SP and RR are more severe and frequent in women than in men [106].

Injury of the blood–brain barrier followed by infiltration of immune cells into the brain and spinal cord occurs in the early stages of MS [107]. Nevertheless, the exact reason behind the immune reaction against the nerve fibers remains unclear to date. The major pathological cause is a disturbance of the balance between T cell populations (TCD4 + and Treg) and antigen-presenting cells (microglia) in favor of inflammation following neuronal degeneration. The major subset of autoreactive CD4 + cells (Th1, Th17) seems to be critically involved in MS pathogenesis and its experimental animal model, experimental autoimmune encephalomyelitis (EAE) [108]. Hence, transferring Th_17_/Th_17_ cells to healthy rats causes EAE. Th1 and Th17 negatively affect immune tolerance, leading to inflammatory damage in MS, Th17 by producing inflammatory cytokines such as IL-17a, IL-17F, IL-21, IL-22, and Th1 by secreting TNF-α and IFN-γ [109]. Thus, these cytokines are abundantly expressed in the inflammation area. Although the healing process of neurons occurs naturally, it is not enough to compensate for neuronal loss and prevent disease progression. The current treatment methods (anti-inflammatory drugs, antioxidants, steroidal hormones, and corticosteroids) are available only for RR patients and reduce the number of relapses through immune system suppression [17, 110]. Nonetheless, they cannot stop degradation and replace lost neurons. Moreover, in the case of using this kind of therapies the potential side effects, such as secondary autoimmune disease and increased risk of infection, should be seriously considered [111].

**Fig. 3:**
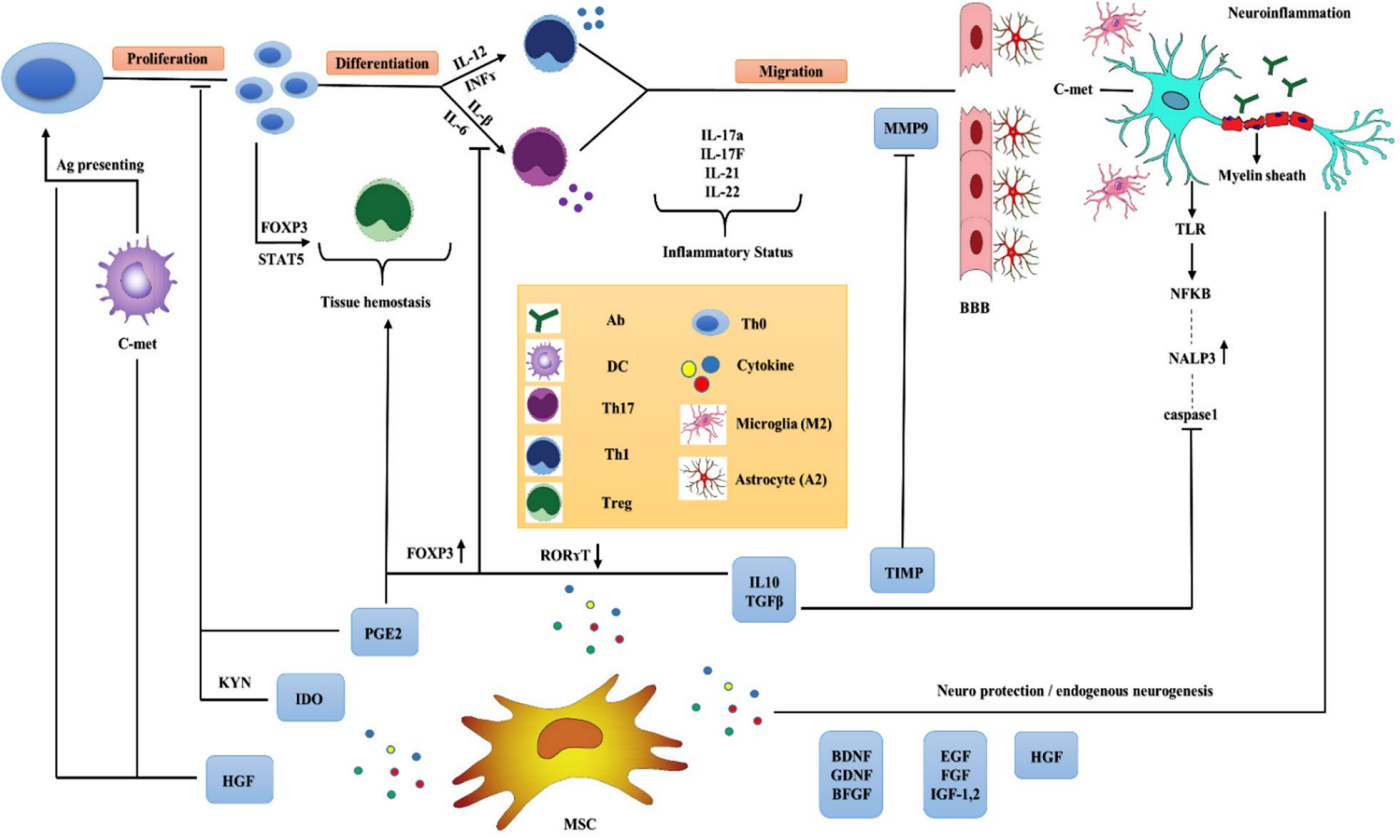
Graphical summary of immunomodulation and neuroprotective effects of MSCs in MS. MSC-derived secretome justifies the unbalanced immune response through secretion of different cytokines and growth factors. These products inhibit the proliferation, differentiation, and migration of Th1 and Th17 to the CNS and increase the Th0 differentiation to Treg instead. MSC secretome also comprises an array of neurotrophic factors which induce endogenous neurogenesis; this compensates lost neurons. MMP9: matrix metalloproteinases9, TIMP: tissue inhibitor of metalloproteinase, IL: interleukin, IFN-γ: interferon-gamma, PGE2: prostaglandin E2, IDO: indoleamine 2,3-dioxygenase. HGF: hepatocyte growth factor, BDNF: brain-derived neurotrophic factor, GDNF: glial cell-derived neurotrophic, BFGF: basic fibroblast growth factor, IGF: insulin-like growth factors, VEGF: vascular endothelial growth factor, KYN: kynurenine, FOXP3: forehead box P3, STAT5: signal transducer and activator of transcription 5, C-met: hepatocyte growth factor receptor, BBB: blood–brain barrier. The images depicted in the figure are designed by authors

As mentioned previously, the role of immune cells is undeniable in MS pathogenesis. Accordingly, targeting these immune cells and recovering from immune hemostasis can be an effective and promising strategy for treating this disease. MSC-derived secretome can justify the unbalanced immune response in MS through different mechanisms. These factors also exhibit neuroprotective effects that inhibit neuronal degeneration after the disease's progression. The following paragraphs describe different mechanisms induced by MSC secretome (Fig. 3).

MSC-derived cytokines can induce Th0 differentiation to other T cell subsets. IL-10 and TGF-β are competent representatives of these cytokines. Following investigations of the underlying mechanisms, it was observed that co-cultured BM-MSC with CD62L^high^ CD44^low^ CD4 + CD25^low^ T cell population reduced differentiation of Th0 toward Th17 through downregulation of the RORγT-related signaling pathway. Researchers have demonstrated that the increase in IL-10 secretion might play a key role in this action, so that IL-10 neutralization could significantly restore the Th17 population [112]. Svobodova et al. co-cultured the alloantigen-stimulated spleen cells with TGF-β releasing MSCs to examine the effect of MSC-derived TGF-β on the naïve T cell differentiation. They confirmed that TGF-β reciprocally regulated Th17 development and its secretory cytokine, IL-17, as well as two crucial transcription factors, Foxp3 and RORγT []. As a result, inducing Th0 differentiation into the Treg cell population instead of Th17 is known as another chief function of the mentioned cytokines, resulting in changing the Treg/Th17 ratio in favor of tissue hemostasis. Tregs also suppress the DC-induced Th_17_ differentiation [113].

Inhibition of T cell proliferation is another critical mechanism induced by MSC mediators. HGF exerts its indirect T cell anti-proliferative role by regulating c-met receptors in antigen-presenting cells, especially DCs [76]. However, the direct suppression effect of this receptor on Th17 remains unclear. In addition to the HGF functional assessment, another study designed by Bai et al. demonstrated improved memory deficit and functional recovery following the infusion of HGF-induced MSC secretome to EAE mouse models. Their results revealed that HGF is a pleiotropic cytokine with neurotrophic and immunomodulatory effects [114]. PGE2, as another potent immunomodulatory product of MSC, can prevent the Th1 and Th17 proliferation and secretion of their inflammatory cytokines [115]; meanwhile, it could be controversial depending on lymphocyte maturation status [116]. IDO specifically released by human MSCs, exerts an anti-inflammatory role by inducing the production of kynurenine, a toxic metabolite against T cell proliferation and apoptosis [117].

MSC-derived cytokines inhibit T cell migration through the BBB. In addition to preventing the antigen presentation to T cells and limiting their proliferation capacity, MSC secretomes have other functional methods to inhibit neuronal degradation and disease progression in MS. Autoreactive T cells can break the BBB and migrate to the CNS neurons using matrix metalloproteinase (MMP)-9, a well-known proteolytic enzyme involved in the MS and EAE pathogenesis, which lyses the myelin sheath [118]. Considering these data, inhibiting MMPs is a potential concept to develop a novel therapeutic method for neuroinflammatory diseases, especially MS [119]. Researchers have indicated that MSC secretomes could relieve disease severity by preventing MMP activity [120, 121]. In this regard, another study introduced MSC-secreted tissue inhibitors of metalloproteinase (TIMPs) as a potent inhibitor of MMP [122].

The immunomodulatory effect of MSCs can be partly explained by inflammasome inactivation. NALP3 is a well-known inflammasome involved in various autoimmune diseases, including MS [122]. This elucidates the therapeutic effect of PDLSC-derived secretome in MS, acting similarly to the conventional immunosuppressant drugs, such as interferon-beta (IFN-β), and suppressing the NALP3 inflammasome and the NFkβ signaling pathway via secreting cytokines such as IL-10 and TGF-β [123]. The inhibitory effect of IL-10 on activated macrophages via inhibiting NALP3 was previously confirmed [124]. It was reported that the main effect of IFN-β is immunomodulation and enhancement of BDNF levels [125].

In conclusion, MSCs can induce neuroprotection and endogenous neurogenesis at injury sites by secreting neurotrophic mediators to improve neuronal survival. Since the levels of neurotrophic factors are dramatically reduced in the CNS of MS patients, increasing their rate or at least maintaining their physiologic level seems to be a valuable therapeutic option [126].

### Parkinson's disease (PD)

Following AD, PD is known to be the most prevalent neurodegenerative disease, discovered by James Parkinson in 1817. It affects approximately 1%-3% of the population above the age of 60 [1]. Among different factors involved in PD pathogenies, including disruption of the protein cleanup pathway, mitochondrial dysfunction, oxidative stress, and genetic mutation, the accumulation of α-synuclein protein in the Lewy bodies (LBs) is the most well-known pathological feature in the disease development [127]. Following damage to the mitochondria, the toxic effect of LBs induces apoptosis in dopaminergic (DA) neurons, specifically in the substantia nigra [128]. The progressive degeneration of DA neurons develops several symptoms of motor dysfunction, including postural instability, bradykinesia, and rigidity [129].

The current accepted standard therapy for PD is levodopa (L-DOPA). Though it alleviates the major symptoms, its dosage should be increased due to the inability to replace DA neurons, thus increasing its side effect. Additionally, its short half-life necessitates the use of other treatment methods [130]. To the best of our knowledge and according to the US National Institutes of Health website (http://www.clinicaltrials.gov), there are just two studies that reported cell-based treatment for PD. Li et al. reported that after fetal mesencephalic dopaminergic neuron transplantation to two PD patients, the neurons survived for over 10 years. However, it was revealed that the newly engrafted neurons were also affected by pathological conditions following α-synuclein accumulation in LBs [131]. In contrast, in seven patients who had BM-MSC autologous engraftment to the sub-lateral ventricular zone via surgery, three of them showed significant improvements, and two others had their medicine dosage reduced; no evidence of tumor growth was observed in the MRI after 12–36 month. Other papers focused mainly on animal models of PD [132]. As the preparation step of stem cell therapy is time-consuming and the procedures for keeping the engrafted cells alive in the transplantation zone are difficult to manage, secretome-based therapy for PD has attracted remarkable attention over the past few years.

The findings of several studies on animal models revealed that MSC-derived secretomes hold significant potential for PD treatment. Different strategies, such as inducing neuronal differentiation, increasing proliferation, raising density, and increasing neuronal viability, were reported by different research groups as having effectively improved motor dysfunctions. The discovery of the regenerative and protective effects of MSC secretomes on DA neurons has attracted a great deal of scientific attention to this new therapeutic aspect. Teixeira et al. revealed that injecting secretomes into rats’ dentate gyrus enhanced the endogenous proliferation of hippocampal neurons after seven days. They incubated human umbilical cord perivascular cells-derived secretomes with human telencephalon neural progenitor cells for five days and reported that neuronal differentiation and density increased in both mature and immature cells. NGF and FGF-2 levels increased simultaneously in that region [77]. Sakane and Miyamoto introduced CHD2 as an important modulator molecule in DA neuron differentiation and proliferation through the regulation of the Wnt-β-catenin signaling pathway [133].

Some evidence has confirmed that MSC secretome-mediated functional recovery in DA neurons. In mouse models of PD, DA neurons progressively degenerated, and accordingly, motor coordination was impaired. The researcher verified the motor performance improvement following injecting the MSC-derived secretomes compared to the control group. However, its effects gradually decreased as time passed, probably due to local consumption. They also showed that the tyrosine hydroxylase (TH) + neurons increased in the test groups compared to those in the control group [57]. The increased number of TH + neurons could be considered one of the factors involved in improving functional balancing. Following the detection of possible contributing factors in this action, Fábio and Teixeira introduced 21 proteins, namely BDNF, VEGF, IL-6, GDNF, cystatin-C, porcine epidermal growth factor (PEGF), galectin-1, heat-shock protein (HSP)-27, TRX1, UCHL1, semaphorin 7a (SEMA 7A), stromal cell-derived factor (SDF)-1, clustrin, CypA, CypA, CypC, DJ-1, cadherin (CDH)-2, PRDX1, UBE3A, and MMP2 by mass spectrometry [134]. Similarly, Cerri et al. confirmed GDNF, BDNF, VEGF, and IL-6 as the most effective molecules in restoring the functional balance of the dopaminergic system [135].

Strong evidence has emphasized the effect of neuronal growth factors on neuronal viability following secretome therapy. It was reported that BDNF expression is reduced in the substantia nigra pars compacta (SNC) in the early stages of PD [136]. Researchers have also introduced this molecule as an essential factor in the development and plasticity of DA neurons preventing neurodegeneration and increasing neuron viability and survival [137, 138]. Hence, knocking down the BDNF gene is associated with increased susceptibility and neuronal loss in DA neurons [139]. GDNF is another neurotrophic molecule involved in the viability and survival of DA neurons [140] through upregulating Bcl-x and Bcl-2 anti-apoptotic proteins [141]. As a potent antioxidant, GDNF inhibits ROS-mediated degeneration by increasing antioxidant enzyme activity [142]. IL-6, a scavenger of superoxide radicals, upregulates ROS activity, contributing to DA neurons’ protection [143]. In addition, IL-6 exerted protective effects on DA neurons against MPP + -mediated toxicity [144]. Thus, knocking down the IL-6 gene makes DA neurons more sensitive to methyl-phenyl-tetrahydropyridine neurotoxicity [145].

Several growth factors produced after secretome therapy have been recognized as having either a direct or indirect neuroprotective role in Parkinson’s disease. In addition to increasing neuronal viability, BDNF and GDNF can directly protect the dopaminergic system [146]. The indirect protection is provided by other growth factors like FGF-2 and EGF [147]. Furthermore, certain VEGF subtypes exert protective effects in a dose-dependent manner through both mentioned mechanisms. In response to acute damages to the dopaminergic system, another important neurotrophic molecule, called PDEF [148, 149], has a better functional outcome and easier delivery compared to other therapeutic methods using secreted molecules like GDNF [150]. PDEF exerts its effect by inducing the NF-kB signaling pathway. NF-kB, as a transcription factor, increases the expression of other neurotrophic molecules, such as BDNF and GDNF [], thereby indirectly increasing neuronal viability and survival.

The aforementioned data shed light on the fact that secretomes can relatively compensate for neuronal loss in PD without requiring further cell transfer. Comparing MSC engraftment and secretome to the animal models of PD showed that the secretome is more efficient in increasing the number and density of TH + neurons in the striatum and neural progenitor cells area, which is probably the cause of the low survival rate of the MSCs in these regions [151].

### Preclinical studies and clinical trials

The therapeutic effects of MSC therapy and MSC-derived exosomes in animal models of neurodegenerative diseases are presented in Tables 1 and 2, respectively. While the preclinical results are promising, the safety and efficacy of MSC-derived secretomes in humans have not been confirmed yet.

**Table 2:** Therapeutic effects of MSC-derived exosomes in animal models of neurodegenerative diseases

Disease	Source of exosomes	Therapeutic effect	Refs.	Year
AD	Mouse BM-MSCs	Improved cognitive behavior	[195]	2018
	Human UCB-MSCs	Ameliorated cognitive decline; attenuated neuro-inflammation	[196]	2018
	Human UCB-MSCs	Ameliorates neural impairment	[195]	2018
	Human UCB-MSCs	Alleviated neuro-inflammation; reduced amyloid-beta deposition	[197]	2018
	Human UCB-MSCs	Repaired cognitive dysfunctions	[197]	2018
	Mouse BM-MSCs	Reduced Aβ plaque burden	[198]	2019
	Mouse BM-MSCs	Reduced amount of dystrophic neurites in both the cortex and hippocampus	[198]	2019
	Mouse BM-MSCs	Recovered cognition impairment	[199]	2019
	Not mentioned	Restored cognitive function; increased learning abilities	[200]	2019
	Human, purchased	Enhanced neurogenesis; restored cognitive function	[201]	2019
	Mouse BM-MSCs	Reduced Aβ deposition; improved cognitive function recovery	[202]	202
	Human AT-MSCs	Induced neurogenesis; ameliorated cognitive dysfunction	[203]	2020
	Human UCB-MSCs	Improved cognitive function	[164]	2020
	Human BM-MSCs	Decreased microglia activation; increased dendritic spine density	[204]	2020
	Rat BM-MSCs	Improved in destructive structural changes in the taste buds and their innervations	[205]	2020
MS	Rat BM-MSCs	Decreased neural behavioral scores	[206]	2019
	Human BM-MSCs	Reduced disease severity	[207]	2019
PD	Rat BM-MSCs	Reverted motor phenotype and the neuronal organization	[208]	2017
	Rat AT-MSCs	Induced neuroprotection; Increase neuronal plasticity	[209]	2017
	Rat BM-MSCs	Neuroprotective effect on dopaminergic neuron	[210]	2019
	Human AT-MSCs	Ameliorated neurological complications	[211]	2019
	Human UCB-MSCs	Improved neurogenesis and cognitive function	[212]	2020
	Rat BM-MSCs	Attenuated of dopaminergic neuron loss; Improved dopamine levels in the striatum	[212]	2020
	Rat BM-MSCs	Improved motor function	[213]	2020
	Mouse AT-MSCs	Suppressed autophagy and pyroptosis	[214]	2021
	Mouse AT-MSCs	Promoted angiogenesis of human brain microvascular endothelial cells	[215]	2021
	Human BM-MSCs	Reduced number of α-synuclein inclusions	[216]	2021

*AD* Alzheimer’s disease, *MS* Multiple sclerosis, *PD* Parkinson’s disease, *BM-MSCs* Bone marrow-derived mesenchymal stem cells, *AD-MSCs* Adipose tissue-derived mesenchymal stem cells, *UCB-MSCs* Umbilical cord blood-derived mesenchymal stem cells

Over the past decade, the therapeutic potential of MSC therapy in neurodegenerative diseases has been evaluated in various clinical trials. Table 3 presents all the clinical trials of MSC therapy in AD, PD, and MS patients listed in the US National Institutes of Health Clinical Trials Database (www.clinicaltrials.gov) till September 2022. Although various phase I and II clinical trials were conducted to assess the safety and efficiency of MSC therapy in neurodegenerative disease, the results should be warranted by phase III trials.

**Table 3:** Therapeutic effects of MSC in human models of neurodegenerative diseases

Disease	NCT and Phase	Participants	Stage	Tissue source of MSC	Findings
AD	NCT01297218 (I)	9	Completed	Allogeneic UCB	Feasible; safe; well tolerated
	NCT01696591 (Long-term follow-up of NCT01297218)	9	Unknown	Allogeneic UCB	Not yet published
	NCT02054208 (I/IIa)	45	Completed	Allogeneic UCB	Not yet published
	NCT03172117 (Long-term follow-up of NCT02054208)	45	Unknown	Allogeneic UCB	Not yet published
	NCT03117738 (I/II)	21	Completed	Autologous AD	Not yet published
	NCT04228666 (I/IIa)	24	Withdrawn	Autologous AD	Not yet reported
	NCT02600130 (I)	33	Active	Longeveron	NA
	NCT04855955	1	Available	Autologous AD	NA
	NCT02833792 (IIa)	40	Recruiting	Allogeneic (not mentioned)	NA
	NCT04040348 (I)	6	Recruiting	Allogeneic UCB	NA
	NCT02899091 (I/IIa)	24	Recruiting	Allogenic placenta	NA
	NCT04684602 (I/II)	5,000	Recruiting	Allogenic UCB	NA
	NCT04482413 (IIb)	80	Not yet recruiting	Autologous AD	NA
	NCT01547689 (I/II)	30	Unknown	Allogenic UCB	NA
	NCT02672306 (I/II)	16	Unknown	Allogenic UCB	NA
MS	NCT01745783 (I/II)	26	Completed	Autologous BM	Safety and efficacy
	NCT02326935 (I)	2	Terminated	Autologous AD	NA
	NCT03799718 (II)	20	Completed	Autologous BM	Not yet published
	NCT01854957 (I/II)	20	Recruiting	Autologous BM	NA
	NCT01364246 (I/II)	20	Recruiting	Allogeneic UC	NA
	NCT01377870 (I/II)	22	Completed	Autologous BM	Not yet published
	NCT00395200 (I/II)	10	Completed	Autologous BM	Safe; feasible; positive therapeutic outcomes
	NCT02403947 (I/II)	1	Terminated	Autologous BM	NA
	NCT01895439 (I/II)	13	Completed	Autologous BM	NA
	NCT01933802 (I)	20	Completed	Autologous BM	Safe; well tolerated; minor adverse events included transient fever and mild headaches
	NCT02034188 (I)	20	Completed	Allogeneic UC	Feasible; safe
	NCT02035514 (I/II)	9	Completed	Autologous BM	Not applicable
	NCT02239393 (I)	31	Completed	Autologous BM	Safety and efficacy
	NCT01056471 (I/II)	30	Completed	Autologous AT	Not yet published
	NCT03326505 (I/II)	60	Completed	Allogeneic UC	Not yet published
	NCT01730547 (I/II)	15	Recruiting	Autologous BM	Not yet published
	NCT02495766 (I/II)	8	Completed	Autologous BM	Not yet published
	NCT02166021 (II)	48	Completed	Autologous BM	Well-tolerated; induced short-term beneficial effects
	NCT01606215 (I, II)	21	Completed	Autologous BM	Not yet published
	NCT00781872 (I, II)	24	Completed	Autologous BM	Clinically feasible; safe; induced immunomodulatory effects
	NCT03069170 (I)	50	Recruiting	Autologous BM	Not yet published
	NCT02157064 (NA)	221	Unknown	Autologous AT	NA
PD	NCT01453803 (I/II)	0	Withdrawn	Autologous AT	NA
	NCT02184546	75	Not yet recruiting	Autologous AT	NA
	NCT04064983	Unknown	No expanded access	Autologous AT	NA
	NCT02611167 (I/II)	20	Completed	Allogeneic BM	NA
	NCT00976430	5	Terminated	Autologous BM	NA
	NCT01446614 (I/II)	20	recruiting	Autologous BM	NA
	NCT03550183 (I)	20	recruiting	Allogeneic UC	NA
	NCT04506073 (II)	45	Not yet recruiting	Not mentioned	NA
	NCT04928287 (II)	24	Not yet recruiting	Allogeneic AT	NA
	NCT04995081 (II)	60	recruiting	Allogeneic AT	NA
	NCT03684122 (I/II)	10	Not yet recruiting	Allogeneic UCB	NA
	NCT04146519 (II/III)	50	Recruiting	Autologous (not mentioned)	NA
	NCT05094011 (I)	9	Not yet recruiting	Autologous AT	NA
	NCT04876326 (NA)	15	Recruiting	Autologous AT, Allogeneic UC	NA

*AD* Alzheimer’s disease, *MS* Multiple sclerosis, *PD* Parkinson’s disease, *UCB* Umbilical cord blood, *AD-MSCs* Adipose tissue, *BM-MSCs* Bone marrow, *NA* Not applicable

Despite all preclinical studies demonstrating the efficiency of MSC-derived exosomes in neurodegenerative diseases, their clinical utility has yet to be demonstrated. There are just phase I and II clinical trials initiated to evaluate the safety and efficacy of exosomes secreted from allogeneic adipose tissue-derived MSC in AD patients (NCT04388982).

## Conclusion

MSC-derived secretomes can be used as a promising therapeutic approach in the treatment of neurodegenerative disorders for which no treatment has yet been introduced. This cell-free strategy can solve complications due to MSCs’ migration to the injury site and their differentiation. Because MSCs' beneficial properties are dependent on their ability to deliver their content, MSC-derived secretomes can be as effective as MSCs transplantation in activating the pro-survival and anti-apoptotic signals, leading to improved tissue neuron regeneration. Indeed, MSC-derived secretomes serve as an information transporter from MSCs to neurons.

## Data Availability

Not applicable.

## Abbreviations

CNS: Central nervous system
MS: Multiple sclerosis
AD: Alzheimer's disease
NMDA: N-Methyl-D-aspartate
ISCT: International Society of Cell Therapy
ALS: Amyotrophic lateral
IV: Intravenous
NGF: Nerve growth factor
GDNF: Glial cell-derived neurotrophic factor
BDNF: Brain-derived neurotrophic factor
NT: Neurotrophin
VEGF: Vascular endothelial growth factor
HGF: Hepatocyte growth factor
FGF: Fibroblast growth factor
PEDF: Pigment epithelium-derived factor
IGF: Insulin-like growth factor
TGF-β: Transforming growth factor-beta
IL: Interleukin
IDO: Indoleamine 2,3-dioxygenase
PGE2: Prostaglandin E2
HLA: Human leukocyte antigen
MHC: Major histocompatibility complex
IFN-γ: Interferon γ
TNF-α: Tumor necrosis factor-α
TSG-6: TNF-α-stimulated protein 6
NK: Natural killer cells
BBB: Blood–brain barrier
Aβ: Amyloid-beta
NFT: Neurofibrillary tangles
IFN-β: Interferon-beta
MG: Microglia
AC: Astrocytes
TLR: Toll-like receptor
CCL5: Chemokine ligand 5
ICAM-1: Intracellular adhesion molecule-1
MMP: Matrix metalloproteinase
LB: Lewy body
TH: Tyrosine hydroxylase
